# Smart medical report: efficient detection of common and rare diseases on common blood tests

**DOI:** 10.3389/fdgth.2024.1505483

**Published:** 2024-12-05

**Authors:** Ákos Németh, Gábor Tóth, Péter Fülöp, György Paragh, Bíborka Nádró, Zsolt Karányi, György Paragh, Zsolt Horváth, Zsolt Csernák, Erzsébet Pintér, Dániel Sándor, Gábor Bagyó, István Édes, János Kappelmayer, Mariann Harangi, Bálint Daróczy

**Affiliations:** ^1^ Division of Metabolic Diseases, Department of Internal Medicine, Faculty of Medicine, University of Debrecen, Debrecen, Hungary; ^2^ Aesculab Medical Solutions, Black Horse Group Ltd., Debrecen, Hungary; ^3^ Department of Laboratory Medicine, Faculty of Medicine, University of Debrecen, Debrecen, Hungary; ^4^ Department of Dermatology, Roswell Park Comprehensive Cancer Center, Buffalo, NY, United States; ^5^ Center of Oncoradiology, Bács-Kiskun County Teaching Hospital, Kecskemét, Hungary; ^6^ Central Medical Department, Synlab Group (Synlab Hungary Ltd.), Budapest, Hungary; ^7^ Central Laboratory, St. John’s Hospital, Budapest, Hungary; ^8^ Laboratory of Immunology, Synlab Budapest Diagnostic Center, Budapest, Hungary; ^9^ Department of Artificial Intelligence and Systems Engineering, Faculty of Electrical Engineering and Informatics, Budapest University of Technology and Economics, Budapest, Hungary; ^10^ Evidia MVZ Radiologie, Nürnberg, Germany; ^11^ Department of Cardiology, Faculty of Medicine, University of Debrecen, Debrecen, Hungary; ^12^ Artificial Intelligence National Laboratory, Institute for Computer Science and Control (SZTAKI), Hungarian Research Network (HUN-REN), Budapest, Hungary

**Keywords:** blood test analysis, chronic diseases, rare diseases, machine learning, prevention and control, classification

## Abstract

**Introduction:**

The integration of AI into healthcare is widely anticipated to revolutionize medical diagnostics, enabling earlier, more accurate disease detection and personalized care.

**Methods:**

In this study, we developed and validated an AI-assisted diagnostic support tool using only routinely ordered and broadly available blood tests to predict the presence of major chronic and acute diseases as well as rare disorders.

**Results:**

Our model was tested on both retrospective and prospective datasets comprising over one million patients. We evaluated the diagnostic performance by (1) implementing ensemble learning (mean ROC-AUC.9293 and mean DOR 63.96); (2) assessing the model's sensitivity via risk scores to simulate its screening effectiveness; (3) analyzing the potential for early disease detection (30–270 days before clinical diagnosis) through creating historical patient timelines and (4) conducting validation on real-world clinical data in collaboration with Synlab Hungary, to assess the tool's performance in clinical setting.

**Discussion:**

Uniquely, our model not only considers stable blood values but also tracks changes from baseline across 15 years of patient history. Our AI-driven automated diagnostic tool can significantly enhance clinical practice by recognizing patterns in common and rare diseases, including malignancies. The models' ability to detect diseases 1–9 months earlier than traditional clinical diagnosis could contribute to reduced healthcare costs and improved patient outcomes. The automated evaluation also reduces evaluation time of healthcare providers, which accelerates diagnostic processes. By utilizing only routine blood tests and ensemble methods, the tool demonstrates high efficacy across independent laboratories and hospitals, making it an exceptionally valuable screening resource for primary care physicians.

## 1 Introduction

Chronic workforce shortages, unequal distribution of healthcare professionals, and rising labor costs present significant challenges for most healthcare systems worldwide. In recent years, technological advancements have aimed to mitigate these pressures. The broad adoption of digital databases, modern diagnostic testing and screening, and the expansion of digital communication channels created the opportunities to redistribute the workload of skilled medical professionals. This technological transformation of the available tools of healthcare systems combined with pandemic-induced restrictions, has led to the accelerated transition and acceptance of virtual medicine. Laboratory follow-ups, in particular, offer an ideal environment for virtual healthcare encounters. Routine tests can be performed in remote, controlled environments, convenient for the patient, and communication between healthcare providers (HCPs) and the patient can be performed via digital platforms.

Despite this, the diagnostic process remains largely reliant on the clinical judgment of HCPs, based on the diagnostic test results. While clinical pathology/laboratory medicine physicians perform and report these tests, the direct communication with HCPs is typically limited to critical or emergency situations, leaving most non-emergency cases without in-depth interpretive support. This creates a gap where a more comprehensive and in-depth analysis of laboratory results could greatly aid HCPs in reaching accurate diagnosis.

However, such detailed evaluation support is currently impractical at the clinical-pathologist level due to the sheer volume of patients and tests conducted. Therefore, other means of evaluating laboratory results are sorely needed.

Diagnostic errors and delays, which occur in 15%–20% of all patient-doctor encounters (1, 2) are significant concerns in modern medicine, contributing to delayed diagnoses and misdiagnoses, making them the sixth leading cause of death in the United States (3). Interestingly, while diagnostic errors persist, laboratory error rates are exceptionally low, estimated at just .33% (ranging from .1% to 3.0%) (4); indicating that laboratory test results are reliable and high-quality source of information that could be leveraged to improve diagnostic processes through technology such as artificial intelligence (AI).

Blood tests are among the most common and cost-effective diagnostic tools in medicine. They provide vital information on a wide range of physiological conditions, from routine screenings to complex disease monitoring. However, interpreting the growing number of specialized tests can be challenging due to human limitations, particularly when test results are borderline or inconclusive. Diagnostic errors in medicine are of a complex origin and are mostly classified as no-fault, system-related, or cognitive errors. Cognitive errors are primarily due to the false processing of available information and often account for the most severe misdiagnoses (2). Moreover, many rare diseases are notoriously difficult to diagnose, often resulting in delayed recognition. For instance, a study of late-onset Pompe disease showed that 48% of patients were incorrectly diagnosed in the past, with an increased mean time to diagnosis of 10.5 vs. 2.5 years (from symptom onset) (5).

Blood tests were chosen as the focal point for this AI application due to their ubiquity, low cost, and vital role in diagnostic processes. Furthermore, their high reliability presents an ideal foundation for applying machine learning algorithms to enhance diagnostic accuracy and aim to provide evidence that such tools will transform clinical diagnostic processes, improving diagnostic accuracy and enabling earlier diagnosis. Our objectives were to

1. Train and test our ensemble learning model to diagnose rare and common chronic diseases on widely available, low-cost blood tests.
2. Evaluate the diagnostic performance of the AI and the possibility for earlier diagnosis.
3. Implement our AI-based diagnostic support tool in practice and evaluate in-practice performance vs. performance on retrospective data.

## 2 Related work

Artificial intelligence (AI) has the potential to revolutionize the interpretation of blood tests and improve diagnostic accuracy. Since the emergence of deep learning technologies in 2012, AI has rapidly advanced within healthcare, with substantial applications in areas such as medical imaging and image-based diagnosis. More recently, additional field genome interpretation and biomarker discovery, and patient monitoring have also gained increased importance (6). However, in laboratory medicine, as reviewed in Santos-Silva et al. (7), AI advancements have been focusing on narrow diagnostic areas, and include only one or few specific laboratory parameters (7). These AI developments included the evaluation of bacterial infection on hospital admission (8), predicting bacteremia in maternity patients (9), classifying outcomes in malaria infection based on hematological parameters (10), or supporting clinical pathologists in flow cytometry evaluations (11, 12), and detection of Alzheimer's disease based on specific data about the state of the brain and patient's metadata (13). The primary disadvantage of these models is the narrow diagnostic focus, which makes them less efficient to implement and utilize on large numbers of patients and conditions. For a detailed description and comparison of the mentioned literature, see Table 1. We argue that the main challenges in the field of AI-based blood test analysis are:

 • How to achieve robustness despite limitations in the available data?
 • How to identify a general attribute set that can be used across different disease classes that are physiologically connected to the blood work?
 • How to define a set of machine learning methods suitable for preprocessing and inference?

**Table 1:** Comparison of different results of AI based disease or infection detection.

	Summary	Data	Methods	Evaluation	Notes
Santos-Silva et al. (7)	Review of AI methods for common blood tests	n/a	n/a	n/a	A balanced summary of multiple studies of AI disease classification with common blood tests
Rawson et al. (8)	Detection of bacterial infections	160k patients, microbiological and blood tests (CRP, WCC, ALT, BIL, ALL)	SVM	10-fold CV AUC: 0.84 Sens.: 0.89 Spec.: 0.63	Strengths: Early detection (72 h) Limitations: only SVM
Mooney et al. (9)	Detection of bacterial infections during pregnancy and postpartum	129 patients, full blood count, prevalence 3%	Regression Trees, LDA, kNN, SVM, RF, CART	70–30 split Best method CART: Sens.: 0.28 Spec.: 0.94 PPV: 0.13 NPV: 0.97	Strengths: strong machine learning methodology Limitations: small dataset, no AUC results
Morang et al. (10)	Detection of different Malaria	2,207 patients, hematological parameters (RBC, Platelet, Lymphocyte)	3 layer ANN, regression models (PLS, MARS)	Acc.: 80%/96% (uncomplicated/severe Malaria) AUC: 0.866/0.983 F-score: 0.747/0.947	Strengths: very high performance Limitations: no boosted trees, limited size of patients
Lu et al. (11)	Diagnosis of immunological disorders	379 patients, cytometry (3-tube, 10 color flow panels) with 21 antibodies	LR, DeepFlow^TM^	Correlation between manual analysis and AI assisted model, *r* > 0.9	Strengths: high correlation Limitations: highly specific and small data
Alcazer et al. (12)	Acute Leukaemia subtype detection	1,410 diagnosed patients, 19 (mainly) routine parameters are used	GLM, Naive Bayes, RF, XGB	10-fold CV, best method XGB: AUC: 0.67–0.97	Strengths: high performance, wide variety in data due to different sources, strong methods Limitations: authors compare only different subtypes
Loveleen et al. (13)	Detection of Alzheimer's disease	150 patients (age 60–96y), meta data (gender, education, mental state), MRI evaluation	SVM, MLP, LR, Decision Trees (DT), kNN	AUC: 0.72–0.79 (best SVM) Acc.: 80–100% (best DT) Recall: 0.60–0.79 (best DT)	Strengths: strong machine learning, good performance Limitations: authors evaluate their models on the training set, limited attribute set

In this paper, we primarily focus on these questions with the intention to create a solution to evaluate routine blood tests across a broad spectrum of diseases, thereby decreasing the human professional workload within the interpretation of screening tests and providing a broadly used method for risk mitigation of medical decisions and differential diagnosis.

## 3 Methods

The development leveraged 15 years and 1.3 million complete, uncleaned, but anonymous patient medical record data (2000–2015, directly from the HIS - Hospital Information system) in cooperation with the University of Debrecen Clinical Center (UDCC), County Hospitals of Szabolcs-Szatmár-Bereg (CHSSB) and UDCC's contractual software developer partner (Aesculab Medical Solutions - Black Horse Group Ltd.). The sites involved in the research were the following: UDCC, the András Jósa University Hospital, and additional smaller hospitals in neighboring cities, five hospital units, and their outpatient centers (all together referred to as CHSSB). Table 2 shows the diseases, the number of patients included in the retrospective study, and the descriptive statistics for each group.

**Table 2:** Descriptive statistics of the ensemble training set.

	Patient # (*N*)	Patient rate (%)	Patients’ age (years)	Male to female ratio	Patients 18 + yo.
All patients incl. all healthy individuals	1,163,723	100.00%	38.12 ± 23.37	44.64%	77.37%
Thyroid diseases	73,143	6.29%	49.32 ± 18.37	16.70%	94.85%
Liver diseases	42,404	3.64%	54.23 ± 15.39	60.56%	98.24%
Kidney diseases	54,214	4.66%	61.08 ± 20.62	43.59%	96.17%
Inflammatory bowel diseases	41,954	3.61%	30.91 ± 26.96	45.36%	59.08%
Lipid metabolism disorders	79,589	6.84%	59.16 ± 13.24	45.05%	99.53%
Nutritional anemias and disorders	16,669	1.43%	44.90 ± 26.69	33.87%	79.20%
Anemias other than nutritional	76,790	6.60%	48.11 ± 29.27	44.70%	77.47%
Diabetes mellitus (type 1 and 2)	75,028	6.45%	61.33 ± 15.07	47.14%	97.62%
Systemic autoimmune disorders	18,070	1.55%	53.20 ± 17.13	21.05%	96.22%
Gallbladder and pancreatic disorders	49,357	4.24%	57.50 ± 17.85	36.82%	98.46%
Disorders involving immune mechanism	10,757	0.92%	22.20 ± 23.06	50.20%	44.89%
Cardiovascular diseases	238,062	20.46%	60.58 ± 15.44	45.52%	99.34%
Leukemias and lymphomas	7,167	0.62%	59.14 ± 18.34	49.15%	94.83%
Malignancies of the digestive system	18,007	1.55%	65.90 ± 12.30	55.79%	99.89%

Patients with multiple diseases (comorbidities) were included in each disease category.

Due to the cardinality of the patients, and the variability of the contextual factors regarding age, sex, occupation, and availability of the healthcare system, we developed a framework to preprocess the data. As a result, we managed to identify disease groups where we met all the necessary conditions to apply machine learning while achieving a reliable, reproducible, and scalable system. The dataset we used contained data from multiple hospitals and laboratories. One of our biggest challenges was to make the data comparable and to find robust models that are as insensitive to data from different sources as possible.

### 3.1 Creating the set of disease cases

**Figure 1:**
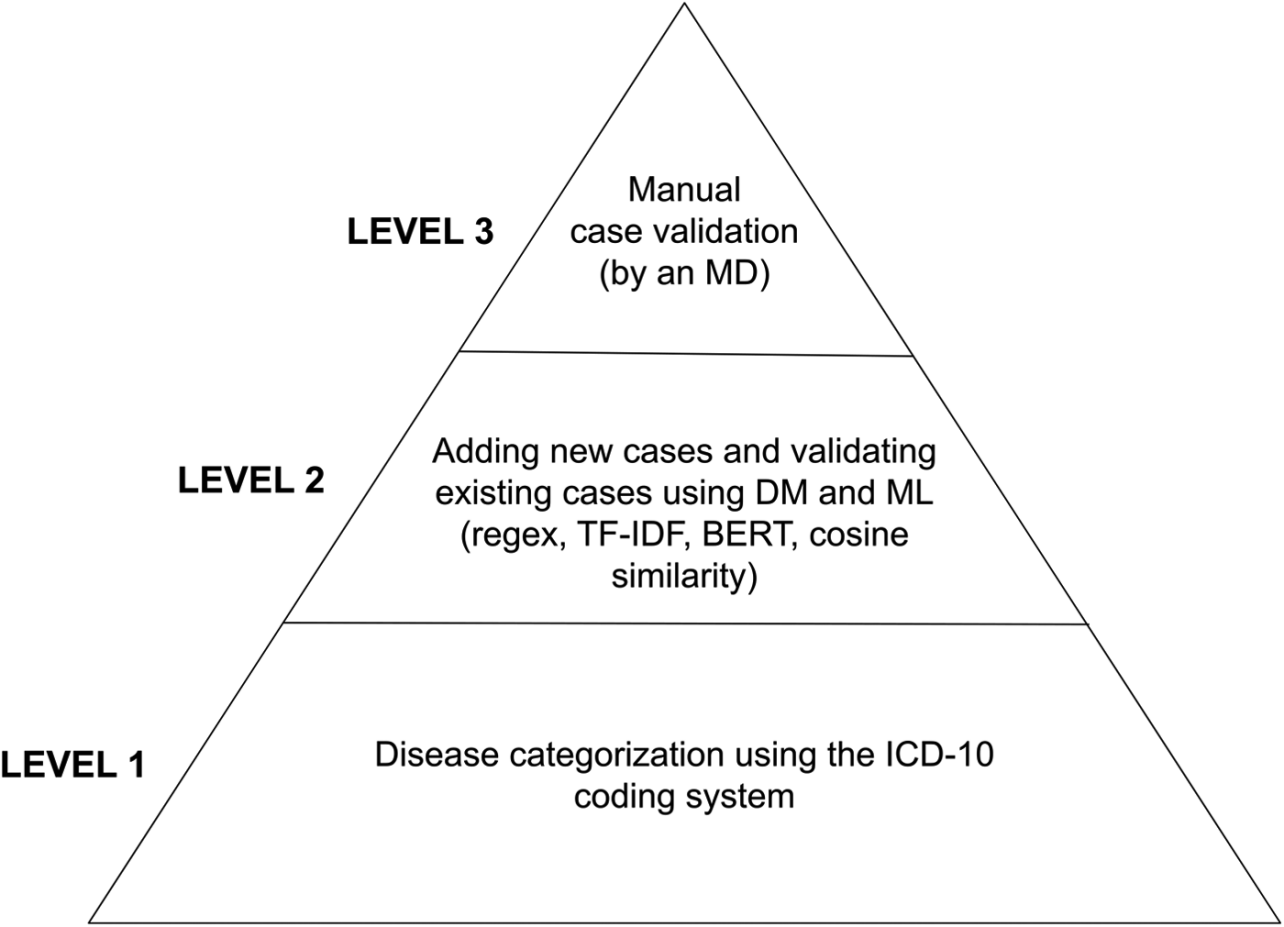
Layers of validation procedures starting from mass categorization based on HIS data and timely assignment (bottom of the pyramid) toward refinement with modern data mining tools and manual validation. BERT, bidirectional encoder representations from transformers; DM, data mining; MD, medical doctor; ML, machine learning; TF-IDF, term frequency-inverse document frequency.

**Figure 2:**
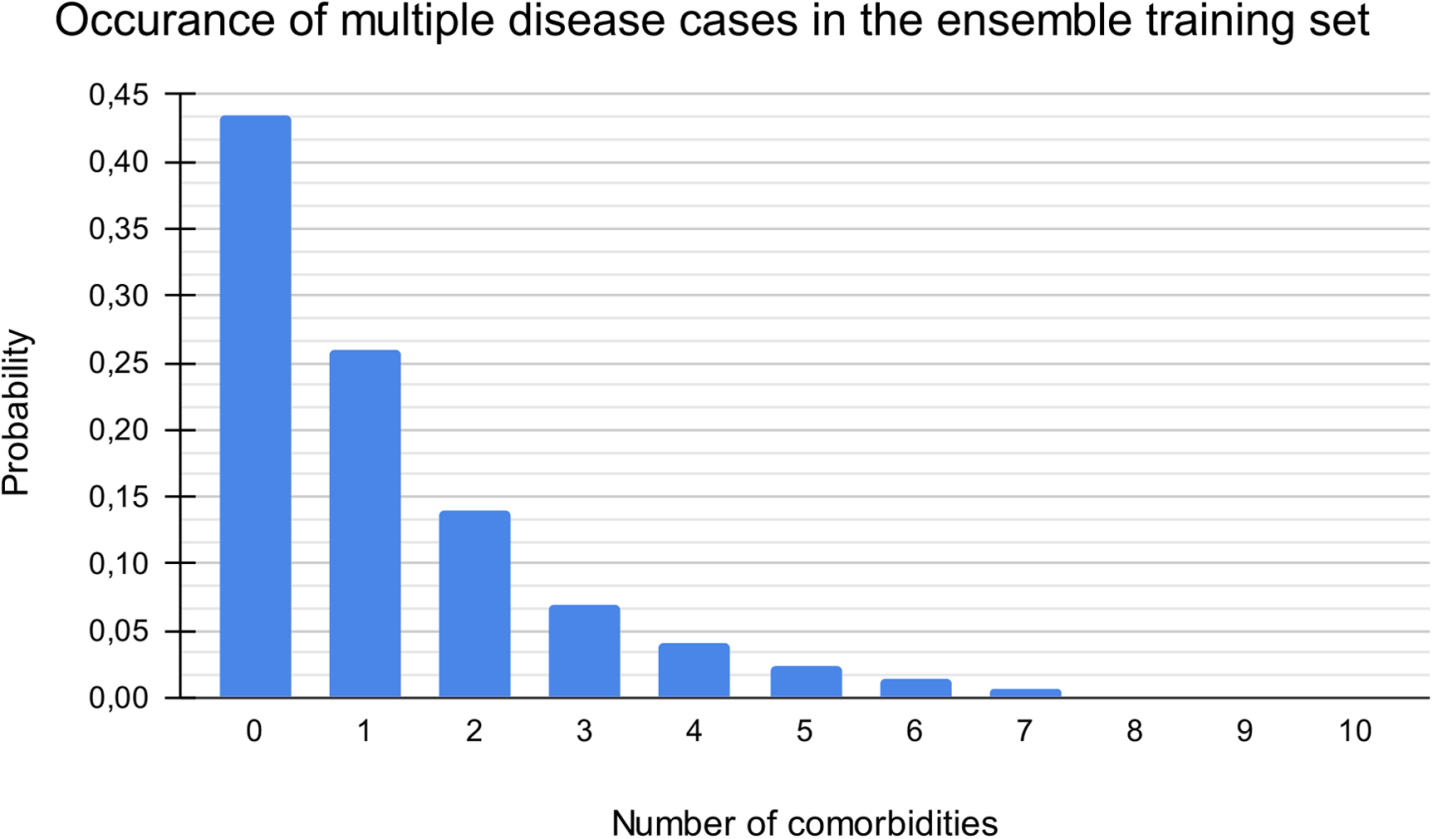
Distribution of patients by number of comorbidities. The maximum number of simultaneous diseases was ten (out of the 14 categories). Zero comorbidity indicates a healthy patient (without suspected or reported diagnosis).

Identifying valid disease labels before applying machine learning methods was essential for the disease classification software. This labeling procedure had to be primarily machine-driven due to the vast amount - tens of thousands - of medical cases. We used a multilevel structure of mainly machine-led and partly manual labeling validation procedures. The first layer of disease categorization was the ICD-10 coding within each patient visit record, which is mandatory in the Health Information System (HIS) database. Each medical case had at least one applied disease code in the HIS. Therefore, we labeled each case with our defined disease group, disease, or sub-disease labels based on the ICD-10 code(s) of the given case in the HIS. Records from the early 2000s used the ICD-9 framework, but the transition to the ICD-10 already occurred at the institutional level before accessing the datasets; thus, we did not need to address any transitional issues. Both the primary and additional diagnoses were labeled. As a subsequent layer, we leveraged available textual anamnestic data to (1) remove non-similar miscategorized cases and (2) find additional disease cases that missed the proper ICD coding. We used the classic data mining library of regular expressions (regex), the statistical descriptors term frequency-inverse document frequency (TF-IDF) and bag-of-words (14), and mathematical methods such as cosine similarity matrices and a neural network, the bidirectional encoder representations from transformers (BERT) model (15). This is a crucial step, as this procedure can alter the number of labeled disease cases by 10%–50%. Manual case validation was the last step of the labeling process and is usually considered verification (for diseases of massive case occurrence); however, this process is also a significant step in identifying rare diseases (along with similarity calculations due to their heavy underdiagnosis), see Figure 1. Each medical case/patient can have multiple conditions and thus may be categorized into multiple disease categories. Each patient has one binary condition for each disease group — “yes” or “no”— but a single patient can have multiple “yes” calls for the different diseases, see Figure 2.

### 3.2 Machine learning models

The core elements of the system are disease-specific machine learning models. These models were trained on historical medical data originating from two major hospitals. The models were selected by a validation procedure with four distinct steps. The first objective during selecting the final models was to identify a subset of the available laboratory tests we aim to include. First, we defined candidate laboratory tests, then we dismissed disease-specific tests, e.g., TSH, or expensive tests. Due to statistical reasons, we also excluded rare laboratory tests. In the second phase, we selected a subset of diseases as possible candidates for analysis directly from blood tests. In the third phase, we trained machine learning models for all candidate diseases using the full set of laboratory tests. In the last step, we identified a subset of 70 laboratory tests and a set of disease groups where the models show very high performance with low variance. For a detailed list of blood tests see Table 3.

**Table 3:** List of blood test results used for the AI.

Blood count	WBC, RBC, HGB, HCT, MCV, MCH, MCHC, PLT, RDW, MPV, PDW, NEU, LYM, MONO, EO, BASO
CH metabolism	GLUC, FRUC
Kidney function	UREA, CREA, eGFR
Metabolic breakdown products	UA, tBIL, dBIL
Ions	Na, K, Cl, Ca, Mg, P
Protein parameters	TP, ALB
Inflammatory markers	hsCRP, ESR, MPXI
Iron panel	Fe, TRF, FER
Lipid panel	CHOL, TG, HDL-C, LDL-C
Enzymes	AST, ALT, GGT, ALP, LDH, CK, LIP, AMY
Hormones	sTSH

ALB, albumin; ALP, alkaline phosphatase; ALT, alanine transaminase; AMY, amylase; AST, aspartate aminotransferase; BASO, basophil count; Ca, calcium level; CHOL, cholesterin level; CK, creatine kinase; Cl, chloride level; CREA, creatinine level; CRP, C-reactive protein; dBIL, direct bilirubin concentration; eGFR, estimated glomerular filtration rate; EO, eosinophil count; ESR, erythrocyte sedimentation rate; Fe, iron level; FER, ferritin level; FRUC, fructosamine level; GGT, gamma-glutamyl transferase level; GLUC, glucose level; HCT, hematocrit; HDL-C, high-density lipoprotein concentration; HGB, hemoglobin concentration; K, potassium level; LDH, lactate dehydrogenase level; LDL-C, low-density lipoprotein concentration; LIP, lipase level; LYM, lymphocyte count; MCH, mean cellular hemoglobin; MCHC, mean corpuscular hemoglobin concentration; MCV, mean corpuscular volume; Mg, magnezium level; MONO, monocyte count; MPXI, mean peroxidase index; MPV, mean platelet volume; Na, sodium level; NEU, neutrophil count; P, phosphate level; PDW, platelet distribution width; PLT, platelet count; RBC, red blood cells count; RDW, red cell distribution width; tBIL, total bilirubin concentration; TG, triglyceride level; TP, total protein level; TRF, transferrin level; TSH, thyroid stimulating hormone level; UA, uric acid level; UREA, urea nitrogen level; WBC, whole blood count.

**Figure 3:**
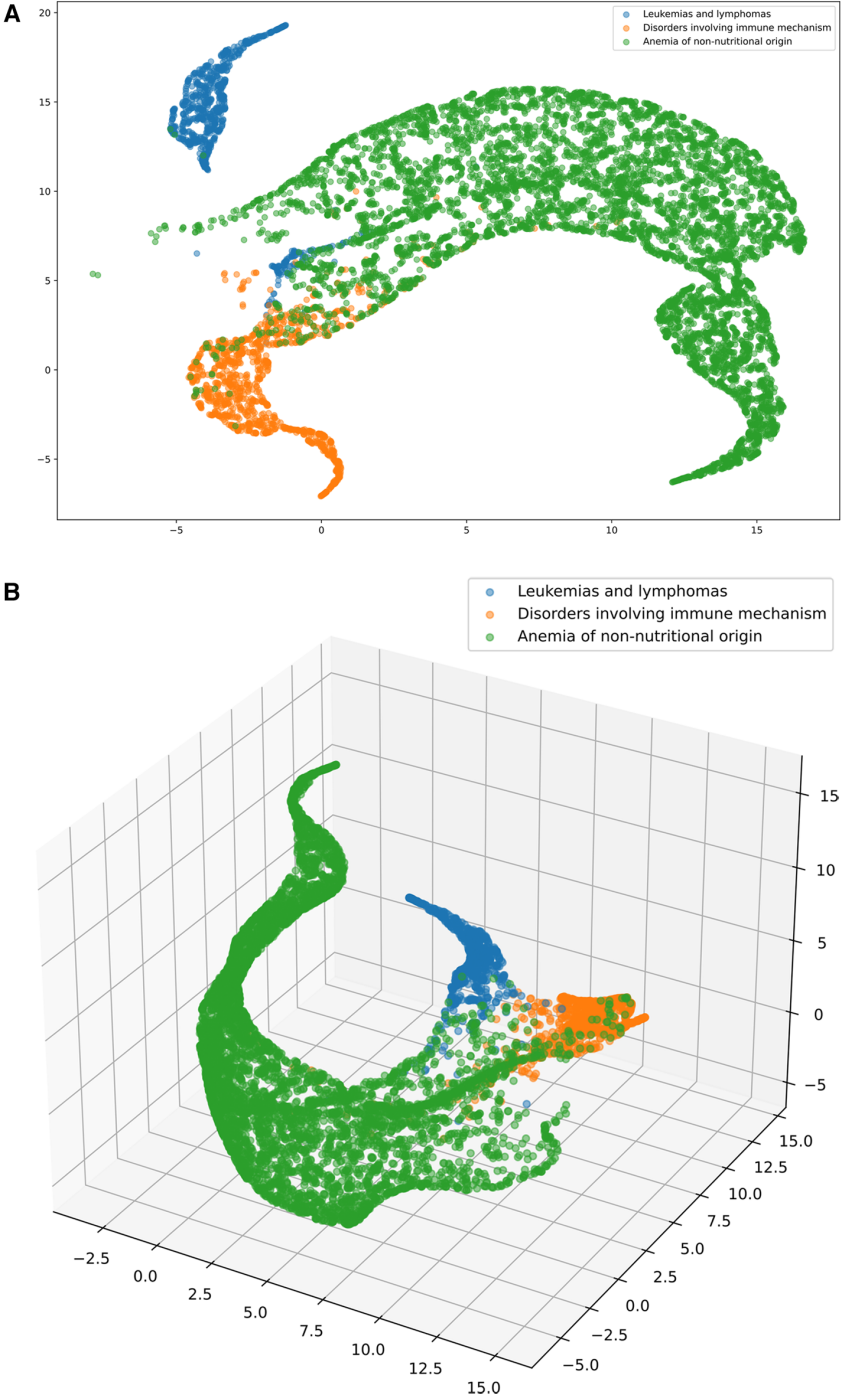
**(A and B)** The software transforms the blood test results (dots) into a multidimensional feature space to separate major hematological conditions (leukemia and lymphoma - blue, immune disease - yellow, anemia of non-nutritional origin - green). 2D (A, on the left) and 3D (B, on the right) visualization via UMAP. The colors indicate the above mentioned medical conditions. We note that we only selected patients with a single confirmed condition (no comorbidities present) for visualization purposes to clearly show where a patient was assigned.

The data shown in the article are based on a random 50–50 split between training and testing for machine learning for software development. The sampling procedure was always repeated 30 times. The data are presented as the average of the different models and train-test data. All the raw data were split for training purposes; the split was made to account for temporal and regional differences in the dataset. For time-based separation, we usually used a five- to eight-year split depending on the participating hospital, partially due to HIS needs (system changes) and to achieve more homogenous ata. The most common time separations were 2000–2005, 2000–2008, 2007–2015, 2006–2010, and 2011–2015. Regional separation was used to separate data from the two leading county hospitals (UDCC and CHSSB), smaller hospitals (CHSSB data), and clinical departments. The training was carried out on the intersections of the separated data, and the resulting performance metrics are the averages of the best-performing models with the restriction that the final ensembles must always contain test- trains on different regional separated data (trained on one hospital/department and tested on another) with different laboratories involved. For more details about the preparation steps for machine learning see Supplementary Appendix A and for a detailed framework structure see Supplementary Appendix B. The disease representation learned by the mathematical models was used to classify patients into one or multiple disease groups. For an extended description of the learned representation and the machine-learning models of disease classification, see Supplementary Appendix C. To visualize the quality of the learned representation, we showed that the patients with different diagnoses align in space using the UMAP (16) framework, see Figure 3A,B.

**Figure 4:**
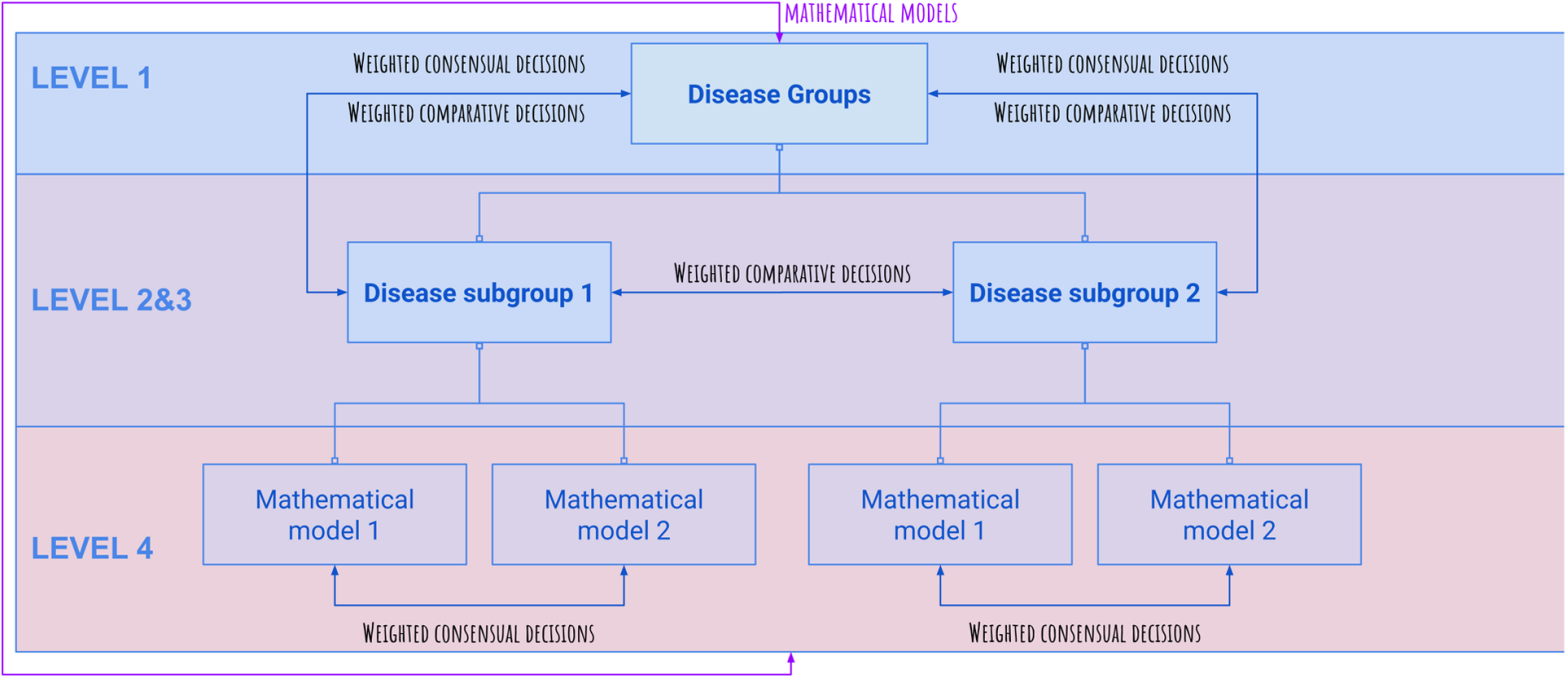
Overview of the framework's tree structure. Comparative (competitive) and consensual decisions are being built up for the final diagnosis of the selected disease group.

Our evaluation procedure included various performance metrics, namely, the receiver operating characteristic (ROC) area under the curve (AUC) (14, 17), diagnostic odds ratio (DOR) (18), accuracy, and sensitivity (14); for their definitions and additional details about the evaluation process, see Supplementary Appendix D. After identifying the best hyperparameters per method, we combined the outputs of the best methods for each classifier with an ensemble layer based on adaptive boosting. The final model for a single disease was a linear combination of the six base machine learning methods of all the disease models, see Figure 4.

### 3.3 Validating in real practice

Validating in a different clinical environment has been carried out with the aid of Synlab Hungary Ltd. and Medisave Ltd. across Synlab's selected private health facilities in nationwide Hungary to test how a routine blood test interpretation and diagnostic support tool may perform in a real environment involving patients and laboratories of various regions. The test was performed in 2023 and included anonymous data of 8,278 patients in an outpatient setting, who agreed to participate at the time of their independent phlebotomy. Every AI-generated medical report has been thoroughly evaluated by at least two medical professionals before the patient received it and in case of any discrepancy between the AI and MD interpretation, the patient acquired written information about the discrepancy and recommendation by the MD. MDs had the opportunity to stop any AI interpretation being sent out in case they considered the discrepancy significant. MDs first evaluated all patients' blood test results for potential diseases, choosing from three possible states for each disease group: “disease is present”, “disease not present”, or “cannot be decided”. Then they reviewed each AI-generated interpretation at both individual disease levels, with “agree”, “disagree” or “cannot be decided” options to choose from, and rated the AI's performance on the report in general on a 1–5 Likert scale. When the AI interpretation showed the risk for a present rare disease, all concerned patients were offered a medical consultation and follow-up testing, out of which 103 ones were carried out (70 for potential rare disease patients and 33 other patients). Significance was always calculated with 95% C. For data protection reasons and retaining anonymity of data processing the AI considered only the actual blood test result of the patient for the prediction in this testing setup, even if previous test results were available. During the training (model build) of the AI, multiple blood tests related to the same person were used for consideration.

## 4 Results

We evaluated the performance of the ensemble system on retrospective data via three main approaches. Details of all the approaches can be found in Table 4 and Table 5. The generated performance statistics describe the models' effectiveness in making aggregated predictive decisions tested on retrospective non-training data. We mapped the continuous zero-one logistic score to a one-to-five scale risk score. Sensitivity shows a monotonic increase in this risk score with respect to the probability of the disease being present. This confirms that our model estimates the risk and that is coherent with the real risk. Finally, early detection scores assess the possibility of evaluating the risk of the analyzed disease groups earlier than the clinically validated diagnosis.

**Table 4:** Average model performance statistics of the used ensemble models^a^.

	Avg. AUC	Avg. DOR	Avg. acc.	Avg. sens.	Avg. specificity	Avg. FPR@0.95 TPR	Avg. positive predictive value (precision)	Avg. negative predictive value
Thyroid diseases	0.9368	73.54	93.03%	79.27%	95.60%	16.88%	0.8664	0.9339
Liver diseases	0.9124	48.32	93.54%	62.59%	98.12%	27.24%	0.7963	0.9593
Kidney diseases	0.9718	112.40	95.01%	80.11%	97.67%	6.43%	0.8582	0.9796
Lipoprotein metabolism disorders	0.9302	49.86	93.82%	80.79%	93.78%	22.62%	0.8584	0.9611
Diabetes mellitus	0.9412	62.13	92.12%	77.46%	96.78%	22.19%	0.8788	0.9405
Non-infected inflammatory bowel diseases	0.8859	72.04	93.48%	42.72%	95,90%	19.26%	0.8075	0.9407
Nutritional anemias	0.9264	64.40	94.66%	68.78%	95.61%	9.15%	0.8476	0.9283
Other anemias	0.9358	50.13	91.95%	79.38%	96.47%	12.59%	0.8460	0.9422
Systemic autoimmune disorders	0.9317	126.39	94.66%	66.38%	98.02%	7.68%	0.8739	0.9498
Gallbladder and pancreatic disorders	0.8847	62.29	93.30%	60.02%	97.88%	32.81%	0.8234	0.9510
Other immune disorders	0.9049	93.89	94.50%	63.65%	99.10%	4.12%	0.8855	0.9540
Cardiovascular diseases	0.9246	34.60	91.13%	79.40%	93.29%	30.57%	0.8420	0.9137
Leukemias and lymphomas	0.9739	376.88	96.83%	81.14%	99.49%	4.82%	0.9516	0.9789
Adenocarcinomas of the digestive sys.	0.9514	251.33	95.48%	62.94%	98.75%	22.42%	0.8222	0.9785

^a^ Average performance statistics of the models used by the software tested on the control population at UDCC (University of Debrecen Clinical Center) and CHSSB (County Hospitals of Szabolcs-Szatmár-Bereg). Performance metrics include Receiver Operating Characteristic (ROC) Area Under Curve (AUC)^8,10^, diagnostic odds ratio (DOR)^12^, accuracy, and sensitivity^8^. Accuracy is defined as the proportion of correctly classified patients in the population. sensitivity rate is the proportion of correctly classified patients with a specific condition in the population. The ROC curve is defined by the point pairs of true positive rates (sensitivity) and false positive rates (1-specificity, where specificity is the proportion of correctly classified patients within the population of the patients without the specific condition) at different threshold settings. The AUC can be interpreted as the probability of classifying a patient with a specific condition with higher confidence than a patient without the condition. The average FPR@ 0.95 TPR is defined as the FPR (false positive rate, 1- Specificity), that is, the percentage of people who are negative but still classified as positive, at which 95% of positive patients are screened (at which sensitivity is 95%).

**Table 5:** Risk score analysis and early detection scores of the used ensemble models^a^.

	Avg. tested recall by risk score					Early detection scores			
	1	2	3	4	5	Avg. LOP (30+)	Avg. LOP (90+)	Avg. LOP (180+)	Avg. LOP (270+)
Thyroid diseases	3.39%	28.82%	58.13%	69.28%	85.42%	−1.15%	−3.35%	−4.70%	−4.66%
Liver diseases	1.37%	8.85%	33.11%	44.68%	74.58%	−3.99%	−5.45%	−6.68%	−10.26%
Kidney diseases	2.53%	29.93%	50.92%	76.87%	91.06%	no loss	no loss	no loss	−2.84%
Lipoprotein metabolism disorders	4.08%	24.28%	39.97%	57.19%	97.91%	−1.63%	−4.13%	−6.52%	−5.19%
Diabetes mellitus	4.51%	24.31%	35.44%	59.01%	94.66%	no loss	−2.10%	−2.17%	−1.93%
Non-infected inflammatory bowel diseases	0.35%	4.79%	11.14%	27.35%	36.86%	−3.33%	−3.30%	−4.97%	−4.89%
Nutritional anemias	2.24%	8.25%	26.05%	41.30%	65.08%	no loss	no loss	no loss	no loss
Other anemias	4.30%	29.22%	46.38%	61.47%	82.20%	no loss	no loss	−3.23%	−5.68%
Systemic autoimmune disorders	2.83%	30.04%	49.89%	72.68%	85.11%	no loss	no loss	no loss	−4.43%
Gallbladder and pancreatic disorders	1.39%	7.36%	14.41%	39.83%	77.60%	−6.04%	−6.10%	−8.72%	−7.61%
Other immune disorders	0.89%	5.29%	8.89%	38.89%	79.31%	no loss	no loss	no loss	−6.96%
Cardiovascular diseases	7.68%	26.56%	40.62%	64.08%	85.38%	−3.17%	−6.57%	−6.52%	−5.96%
Leukemias and lymphomas	1.63%	22.39%	54.90%	70.57%	87.65%	no loss	no loss	no loss	−7.36%
Adenocarcinomas of the digestive sys.	1.05%	12.71%	20.13%	40.61%	54.17%	−1.25%	−3.10%	−5.84%	−20.35%

^a^ Calculated average recall for every risk category score calculated by the software (on UDCC and CHSSB control populations). The higher the risk category score (one to five), the higher the estimated probability of the disease. Meaning of the percentages - given percentage of people receive a proven clinical diagnosis with having a “similar” blood test during the diagnosis period. Early detection scores are calculated based on a set of patients (control group) before they receive a clinical diagnosis. The models are tested to determine if they can provide the received diagnosis earlier only using early blood test results. LOP (loss of performance) scores are calculated between 30 and 365 (30+) and 90–365 (90+) days before the received diagnosis, and average losses are calculated as a ratio of the relevant average ROC-AUC scores vs. the original model performances. “No loss” in cells means that the performance loss of early detection is less than 0.50%, i.e., no significant loss can be detected. Percentage differences below 1.3 ppts between the early detection scores are not significant.

### 4.1 Detection quality

On a diagnostic time scale, the disease appears at an unknown time point, while diagnostic tests lead to an established clinical diagnosis only at a later time point. The tool generally considered only medical cases with a valid diagnosis at the model training stage (only blood tests related to a given diagnosis, either based on HIS data or timely, were used for training). However, we observed that if a broad history of multiple blood tests is available to a person and used for model training, the prediction (ROC-AUC) performance increases significantly, on average +0.0272 ± 0.0213.

**Figure 5:**
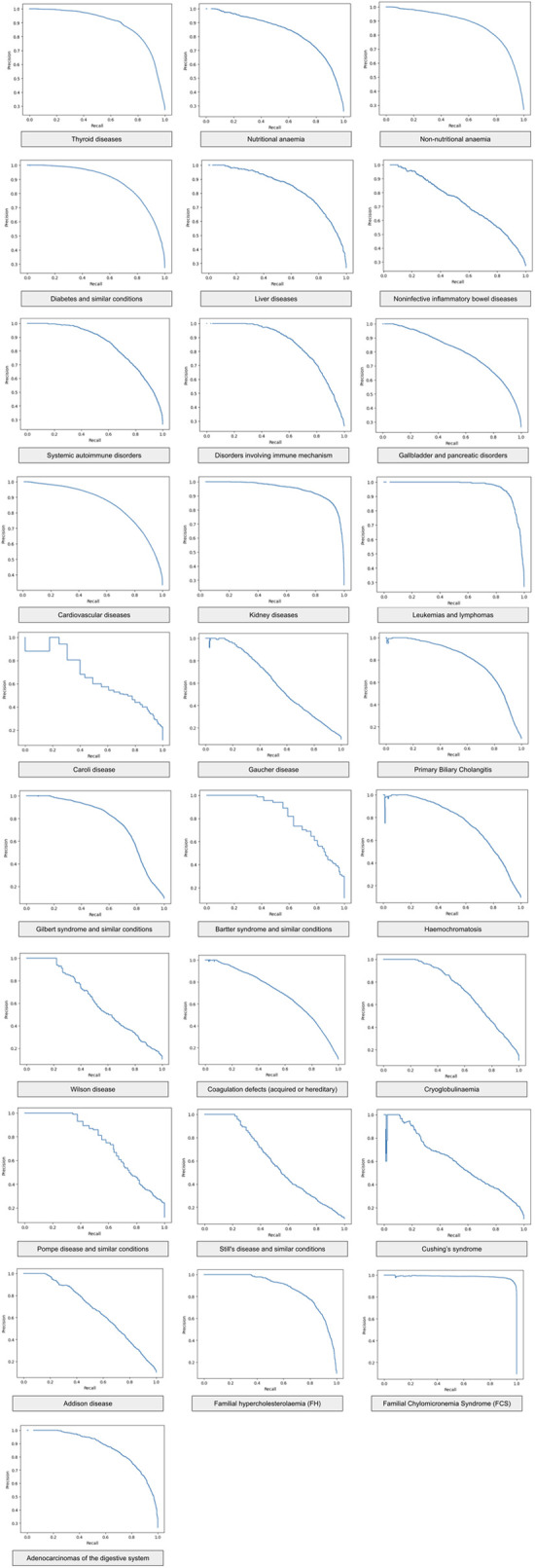
Precision-recall curve of the ensemble method for different diseases.

Table 5 shows that the sensitivity increases monotonously and proportionately with increasing prediction score (projected to one-to-five), which reinforces the hypothesis that the binary classification works well; the higher the prediction score is, the greater the actual chance of disease presence in a single medical case. As the classification system uses “relevant” medical cases (a set of medical cases that results in a specific diagnosis) during clinical disease progression, by using only routine blood tests, see Table 6 for availability of the blood tests in the data, the software can categorize the clinical manifestations of the examined diseases during this period in time (we also carried out detailed testing outside of our time window for early detection). The classification works well for straightforward diagnoses (e.g., kidney, thyroid, or lipid metabolism disorders), hematology (traditionally performed mainly from a complete blood count panel), and complex diseases. We observed a clear presentation pattern for these pathologies during disease progression, where prediction is possible without disease-specific testing. For many disease groups (primarily for more complex ones, i.e., inflammatory bowel diseases and rare diseases), the classification performance may also vary due to the higher rate of underdiagnosis and misdiagnosis. The ensemble method outperformed the individual methods. For the performance of the individual methods see Table 7 and Table 8. Additionally, we report the Precision-Recall curve for all diseases, see Figure 5.

**Table 6:** Percentage of patients with available blood test results on specific days before the establishment of the diagnosis.

	30 + days before	90 + days before	180 + days before	270 + days before
Thyroid diseases	9.3%	7.8%	6.5%	5.4%
Liver diseases	18.2%	14.2%	12.1%	10.6%
Kidney diseases	9.9%	8.1%	6.8%	5.7%
Lipoprotein metabolism disorders	16.9%	14.5%	12.8%	11.4%
Diabetes mellitus	9.7%	7.8%	6.8%	6.0%
Noninfective inflammatory bowel diseases	15.8%	12.3%	10.4%	9.2%
Nutritional anemias	21.3%	16.6%	14.0%	12.1%
Other anemias	32.1%	21.7%	15.5%	11.1%
Systemic autoimmune disorders	5.6%	4.4%	3.2%	2.5%
Gallbladder and pancreatic disorders	19.1%	16.6%	14.8%	13.2%
Other immune disorders	15.5%	11.2%	9.1%	7.8%
Cardiovascular diseases	11.9%	9.9%	8.5%	7.5%
Leukemias and lymphomas	2.1%	1.4%	1.0%	0.6%
Adenocarcinomas of the digestive system	3.2%	2.4%	2.0%	1.4%

**Table 7:** Performance of XGBoost, neural network and SVM models.

	Avg. AUC			Avg. DOR			Avg. acc.			Avg. sens.		
	XGB	NN	SVM	XGB	NN	SVM	XGB	NN	SVM	XGB	NN	SVM
Thyroid diseases	0.9179	0.8704	0.8591	37.19	18.21	89.60	88.4%	81.9%	78.3%	74.4%	79.5%	91.1%
Liver diseases	0.9003	0.8442	0.8486	33.74	13.81	29.11	91.7%	82.2%	80.3%	60.7%	74.7%	82.4%
Kidney diseases	0.8605	0.9326	0.9439	24.62	51.44	65.11	91.7%	89.3%	89.1%	55.6%	83.1%	87.0%
Lipoprotein metabolism disorders	0.8711	0.8857	0.8845	30.33	25.56	21.79	88.7%	82.9%	81.0%	55.2%	86.5%	98.9%
Diabetes mellitus	0.9270	0.8899	0.8906	52.04	23.97	31.57	89.7%	83.9%	82.3%	74.3%	78.7%	86.4%
Non-infected inflammatory bowel diseases	0.8458	0.7700	0.7681	28.80	6.810	11.34	90.7%	77.3%	74.3%	52.3%	63.8%	73.9%
Nutritional anemias	0.8983	0.8347	0.8568	27.79	13.64	15.09	87.6%	80.2%	79.2%	71.4%	75.8%	80.9%
Other anemias	0.9077	0.8637	0.8615	29.57	18.19	17.66	87.5%	81.9%	79.3%	73.8%	80.3%	80.6%
Systemic autoimmune disorders	0.6571	0.8129	0.7769	48.19	10.39	6.84	91.7%	80.6%	75.0%	55.3%	69.3%	92.2%
Gallbladder and pancreatic disorders	0.8638	0.8114	0.8091	34.47	9.02	49.07	90.8%	78.6%	75.7%	55.4%	72.5%	75.4%
Other immune disorders	0.8788	0.8052	0.7938	59.20	12.17	46.32	92.4%	81.6%	77.8%	54.6%	69.3%	78.2%
Cardiovascular diseases	0.8688	0.8926	0.8591	24.22	20.99	14.73	83.4%	82.2%	78.2%	81.0%	82.2%	89.0%
Leukemias and lymphomas	0.9507	0.9272	0.9239	279.9	177.39	356.36	96.1%	90.8%	89.4%	73.5%	78.7%	78.7%
Adenocarcinomas of the digestive sys.	0.9083	0.8626	0.8482	49.48	25.56	42.84	91.7%	83.3%	80.3%	61.2%	78.4%	93.9%

**Table 8:** Performance of logistic regression, elastic network, and Bayes network.

	Avg. AUC			Avg. DOR			Avg. acc.			Avg. sens.		
	logR	ENR	BN	logR	ENR	BN	logR	ENR	BN	logR	ENR	BN
Thyroid diseases	0.8190	0.8781	0.7933	13.59	20.76	17.32	83.4%	80.7%	88.2%	70.5%	87.1%	83.8%
Liver diseases	0.7879	0.8637	0.8108	11.73	17.90	9.48	89.6%	81.7%	84.3%	66.1%	73.7%	75.7%
Kidney diseases	0.8915	0.9487	0.8933	30.40	57.15	22.35	91.7%	89.6%	88.7%	52.5%	85.0%	78.4%
Lipoprotein metabolism disorders	0.8397	0.8966	0.8103	11.23	25.50	13.27	82.5%	82.7%	77.8%	80.9%	88.6%	91.6%
Diabetes mellitus	0.8401	0.9013	0.7923	14.64	30.23	18.30	84.1%	84.2%	77.9%	75.8%	77.8%	86.0%
Non-infected inflammatory bowel diseases	0.7191	0.8117	0.8334	7.68	11.77	19.66	89.5%	78.3%	89.6%	59.3%	70.9%	85.9%
Nutritional anemias	0.8269	0.8681	0.8201	12.84	17.17	10.59	83.9%	80.7%	81.0%	76.5%	76.9%	69.5%
Other anemias	0.8248	0.8716	0.8046	15.21	16.60	9.36	83.7%	80.9%	81.5%	76.6%	78.5%	66.5%
Systemic autoimmune disorders	0.8195	0.7999	0.7606	14.20	8.19	5.37	91.5%	78.2%	89.5%	70.0%	71.0%	79.5%
Gallbladder and pancreatic disorders	0.7325	0.8186	0.7797	8.71	9.99	6.72	89.1%	78.4%	83.3%	64.4%	70.9%	64.3%
Other immune disorders	0.8537	0.8138	0.7519	32.70	19.21	9.60	93.7%	80.2%	84.1%	53.4%	66.8%	64.2%
Cardiovascular diseases	0.8388	0.8623	0.8032	178.5	14.13	11.71	77.1%	80.1%	74.4%	77.6%	83.8%	83.7%
Leukemias and lymphomas	0.8977	0.9336	0.8980	119.49	105.03	110.02	94.5%	90.8%	91.7%	62.1%	79.1%	75.7%
Adenocarcinomas of the digestive sys.	0.7860	0.8777	0.8622	8.66	17.07	18.51	89.5%	85.6%	84.3%	71.0%	79.3%	89.5%

### 4.2 Early detection

When evaluating the possibility of early detection, we used only “non-relevant” medical cases (lab tests), which cannot be directly associated with the studied disease manifestation. Approximately 10% of patients had “non-relevant” cases 30–360 days before the studied diagnosis was established. This number is generally greater for patients with widespread chronic diseases, complications of other (chronic) diseases, or diseases for which diagnostic procedures are less straightforward. This low number could be due to the low participation rate in screening programs and the fact that our data source healthcare institutes mainly provide more advanced care and fewer screening visits. Despite these limitations, thanks to the large sample size, we still gained useful insight into early screening analysis.

### 4.3 Rare disease detection

Among the seven rare disease categories, all had reasonably high ROC-AUC values. Diseases with direct or semidirect markers within routine blood work are of exceptionally high value (dyslipidaemias, hepatic and metabolic diseases, hematologic disorders), which is mainly in line with the findings of common disease groups. Even diseases that require special blood testing (e.g., Wilson disease, Cushing's syndrome, or Addison's disease) show a clear pathologic pattern on routine bloodwork that can be used for educated prediction. Table 9 shows the aggregated average performance of the software for each rare disease.

**Table 9:** Estimated performance for the selected rare diseases.

	Number of patients (*N*) ^a^	Average ROC-AUC score
Familial Chylomicronemia Syndrome (FCS)	21	0.9951 ± 0.0194
Familial Hypercholesterolemia (FH)	459	0.9664 ± 0.0103
Primary Biliary Cholangitis (PBC)	477	0.9449 ± 0.0109
Caroli disease	25	0.8853 ± 0.0971
Gaucher disease	162	0.8836 ± 0.0343
Gilbert's syndrome and similar conditions^b^	2,730	0.9455 ± 0.0079
Bartter syndrome and similar conditions	28	0.8604 ± 0.0841
Hemochromatosis	500	0.9527 ± 0.0134
Wilson's disease	141	0.8627 ± 0.0267
Coagulation defects (acquired or hereditary) ^c^	2,271	0.9139 ± 0.0065
Cryoglobulinemia	189	0.9333 ± 0.0223
Pompe disease and similar conditions	51	0.8990 ± 0.0578
Still's disease and similar autoimmune conditions	117	0.9237 ± 0.0346
Cushing's syndrome	334	0.8601 ± 0.0235
Addison's disease	483	0.8893 ± 0.0163

^a^ Due to the high risk of under or misdiagnosis, rare disease cases were re-evaluated both automatically and manually. As within the scope of the study, there was no possibility to confirm each clinical diagnosis with patient visits, the number of cases is an estimation.

^b^ Gilbert's syndrome is not a rare condition but is very similar to rare, inherited disorders of bilirubin metabolism (e.g., Crigler–Najjar syndrome), which makes it useful to manage as one group.

^c^ Hereditary factor VIII, IX, XI deficiencies, hereditary deficiencies of other clotting factors, hemorrhagic disorders due to circulating anticoagulants, acquired coagulation factor deficiencies.

### 4.4 Importance of different blood tests

Furthermore, with reverse engineering logic, we determined the importance of each laboratory parameter in our AI's diagnosis of each disease group. The data suggest that our AI considers those parameters (laboratory tests) important in confirming or excluding the diagnoses that are also part of the clinical diagnostic definitions in the medical literature, and human medical professionals also consider them in their clinical reasoning. These findings indicate that the AI has revealed similar pathophysiological changes in diseases that have been identified through decades of medical research.

We calculated the importance of individual blood tests in the decision-making of AI in a multistep process in which models were tested multiple times with one or more tests missing from the training. Based on the decrease in the average model performance (ROC-AUC), we created an importance ranking for all the blood tests used: tests whose absence created a greater loss in performance received a higher importance rank. The rank is an average based on the multiple training runs of multiple models. The ranking is described with the score of relative importance, where 100 is the value for the most important test (calculated separately for each examined disease group), and the rest of the tests are represented with a value as a percentage compared to the most important one. High relative importance of a test means that in case that test is missing (e.g., the test was not ordered) then the AI's performance to recognize the disease drops significantly.

The average relative importance, Table 10, of the individual blood test results was measured for all disease groups. The categories show how important the individual test is, on average, for deciding the presence or absence of the disease group.

**Table 10:** The average relative importance of individual blood tests in diagnosing each disease group.

	Very important	Important	Not important
Thyroid diseases	sTSH	Ca, P	PDW, MPXI, LIP, AMY
Liver diseases	AST, ALT, GGT	PLT, GLUC, TG, tBIL, FER	PDW, LDL-C
Kidney diseases	eGFR, Mg, UREA, CREA, hsCRP	CK, sTSH, LDH	FER, TRF, LIP
Lipoprotein metabolism disorders	CHOL, TG	ALP, GLUC, CREA, MPV, LDL-C	Mg, LIP, kBIL
Diabetes mellitus	GLUC, FRUC	TG, eGFR	FER, TRF, kBIL
Noninfective inflammatory bowel diseases	GLUC, FE, AMY, LIP, tBIL	MCH, MCHC, RBC, UREA, ALB, CRP, TRF, GGT, MONO, NEU	Cl, HDL-C
Nutritional anemias	Fe, HGB, MCV	MCH, MCHC, RDW, FER, TRF, CK, RBC, P, tBIL, UREA, Na	–
Other anemias	HGB, HTC, MCH, MCHC	MCV, RDW, Fe, P, NEU	Mg, AMY, LIP, FRUC
Systemic autoimmune disorders	MCHC, RDW, tBIL, CK, MONO, sTSH	MCH, UA, GLUC, HDL-C, HTC, ALP, CHOL, CREA, CRP, MPXI, RBC, K, Mg, GPT, ESR	–
Gallbladder and pancreatic disorders	GGT, LIP, AMY	tBIL, eGFR, kBIL, LDH, ALP, ESR, K, CREA, CK, MONO	PDW, MPXI, Fe, EO
Disorders involving immune mechanism	MCH, LIM, CREA, GGT, RBC, WBC, MCHC, GLUC, CRP	TP, K, Ca, CHOL, MONO, ALT	–
Cardiovascular diseases	ALP, eGFR, GLUC, CHOL, MPV, RBC, CK	UREA, ALB, tBIL, TP, Ca, RDW, MCHC	–
Leukemias and lymphomas	WBC, LIM, RDW, TP, LDH, ESR	MONO, NEU, UA, PLT, BASO	FRUC
Adenocarcinomas of the digestive system	RDW, LIM, CRP, RBC	LDH, ALP, GLUC, GGT, MONO, K	–

Explanation of the columns: (1) Very important: The relative importance of the given blood test for the examined disease group is greater than 70. (2) Important: The relative importance of the given blood test for the examined disease group is between 40 and 70. (3) Not important: The relative importance of the given blood test for the examined disease group is lower than 1. All the other parameters with an average relative importance between 1 and 40 were considered intermediate and are not shown in the table.

### 4.5 Prevalence

When comparing the test results on the data of model build to the validation in a different clinical environment, we see that the results not only deliver the same performance measures but sometimes even outperform them, see Table 11. The disease prevalences were similar to the train population. Anomalies can be found for lipid disorders, cardiovascular diseases, nutritional anemias, and immune diseases. Except for non-inflammatory bowel diseases and cardiovascular diseases, medical doctors fully agreed with well above 95% of the diagnostic proposals of the AI on the individual disease groups. The average report score (on a 1–5 Likert scale) was 4.82. The average scores received by laboratory and clinical practitioners are 4.86 and 4.76, respectively, which means a slight, but significant difference in favor of clinical pathologists when adjudging the performance of the AI.

**Table 11:** General patient statistics and common disease results of the pilot software testing carried out in synlab blood testing facilities across Hungary (all regions) in 2023 with 8,278 outpatients. Every AI-interpreted and evaluated report was cross-evaluated by at least one clinical pathologist and one clinical internist.

	Patients (*N*)	Total patients	Patients (%)	Avg. age	Std. age	Patients (male %)	Avg. report score ^a^	Fully agree (%) ^b^	Est. sens. ^c^	Est. spec. ^c^
Thyroid diseases	487	8,278	5.88%	48.4	±14.7	30.6%	4.81	99.6%	0.99	0.99
Liver diseases	407	8,278	4.92%	51.4	±14.6	46.7%	4.84	98.7%	0.96	0.99
Kidney diseases	316	8,278	3.82%	63.9	±17.5	52.2%	4.88	99.3%	0.96	0.99
Lipid metabolism disorders	3,135	8,278	37.9%	51.1	±13.2	50.0%	4.72	99.9%	0.99	1.00
Diabetes mellitus	373	8,278	4.51%	59.1	±14.5	60.1%	4.79	98.7%	0.98	0.99
Noninfective inflammatory bowel diseases	328	8,278	3.96%	34.6	±11.8	20.9%	4.85	85.4%	0.97	0.98
Nutritional anemias	521	8,278	6.29%	41.3	±15.5	19.2%	4.93	98.8%	0.98	1.00
Other anemias	274	8,278	3.31%	50.2	±18.1	32.1%	4.94	100.0%	0.99	1.00
Systemic autoimmune disorders	17	8,278	0.21%	27.2	±11.4	21.2%	4.91	100.0%	0.93	0.98
Gallbladder and pancreatic disorders	207	8,278	2.50%	58.0	±17.0	45.9%	4.65	96.6%	0.96	0.99
Disorders involving immune mechanism	0	8,278	0.00%	0.00	±0.00	0.00%	n.a.	n.a.	n.a.	n.a.
Cardiovascular diseases	747	8,278	9.02%	59.8	±14.3	48.3%	4.81	94.3%	0.91	0.99
TOTAL	8,278	8,278	100.0%	43.9	±15.0	43.7%	4.82	83.9%	0.98	0.99

^a^ The average evaluation of the reports received by the evaluating medical professional that contained the given predicted disease. The minimum score was 1 and the maximal score was 5.

^b^ The percentage of fully agreed reports is calculated based on the reports that received “agree” evaluation on the given AI predicted disease vs. total reports (other possible evaluations include “don't agree” and “not conclusive”’).

^c^ The sensitivity and specificity are estimated based on a confusion matrix of AI prediction and clinical medical professionals’ patient/report evaluation on the given disease groups.

Interestingly, though, when assessing the decisions on individual disease levels, we see that clinical internists agree significantly more with the AI decisions (98,4% vs. 96,0%). We also measured the time needed for a detailed medical evaluation of all medical reports without (not considering AI-generated reports) and with AI-aided generation reports. We found that with the help of the AI, the report validation time decreased significantly by 50.7% (from 645s to 318s on average).

When assessing potential performance with rare diseases/conditions, Table 12, we checked the estimated prevalence of a certain disease and how it is related to the prevalence in the literature while also appraising the precision based on the medical consultations of real patients. FH and Gilbert cases showed very high estimated precision (95%+), while hemochromatosis also showed an acceptable hit ratio (76%). Other diseases had few or no potential patients, so deeper evaluation was not possible.

**Table 12:** Rare disease predictions during the pilot testing and patient statistics where the software predicted at least 3 out of 5 risk scores. 103 patients participated in medical consultations and checkups where screening precision was estimated.

	Patients with estimated risk (*N*)	Avg. age	Std. age	Patients (male %)	Est. preval.	Lit. preval.	Patients consulted (*N*) ^a^	Est. precision ^b^
Familial Hypercholesterolemia (FH)	39	51.5	±13.5	32.6%	1:212	1:300	25	96%
Primary Biliary Cholangitis (PBC) or similar conditions	13	46.2	±17.1	28.4%	1:637	1:8,000	2	50%
Gilbert's syndrome and similar conditions	313	39.1	±13.2	66.2%	1:26	1:20	20	100%
Hemochromatosis	58	43.8	±18.0	55.2%	1:143	1:300	17	76%
Other rare diseases	31	47.7	±13.3	48.4%	1:267	n.a.	6	n.a.
Other diseases	4,040	48.5	±15.3	42.9%	1:2	n.a.	33	97%

^a^ The number of real patient-doctor visits after the initial AI screening.

^b^ Estimated precision based on the medical consultation. Medical doctors checked the selected patients and received detailed anamnesis and family anamnesis. Specific tests (e.g., genetic testing) were not always available.

## 5 Discussion

The importance of laboratory tests in medical diagnostic processes is undisputed. Recent surveys show that laboratory test results influence 60%–70% of clinical decisions (19), and are embedded in at least 80% of guidelines, which are aimed at establishing a diagnosis or managing a disease (20). The low error rate of laboratory tests makes them a solid base for AI-supported medical diagnostic processes. Numerous studies have shown that laboratory tests can be used to effectively identify chronic diseases or infections (21–25). Similar to these results, our findings demonstrate that machine learning can extend diagnostic capabilities to a broad range of conditions, including rare diseases.

Our findings indicate that AI-aided screening can detect diseases up to six to nine months earlier than current clinical diagnoses, consistent with emerging trends in AI applications for early diagnosis. However, some state-of-the-art methods focus on radiological or imaging-based data, whereas our approach leverages only routine blood test results, which are more readily accessible.

We previously showed that machine learning is ideal for diagnosing rare dyslipidemias (26, 27), but analyzing a wider set of rare diseases shows that many rare diseases may also be ideal candidates for computer-aided screening efforts, even those that usually require (multiple) specific diagnostic tests for confirming the diagnosis. Our initial models were promising to further evaluate the diagnostic opportunities of AI models on laboratory tests, see Table 11. Both prevalence estimation and the estimated precision align with the original calculations and expectations.

We tested the tool's general capability for early screening of supported diseases by evaluating the blood test results of patients who presented regularly at hospitals for screening or other purposes but had not yet received an official diagnosis. As shown in Table 3 in the Results section, routine test-based AI-aided diagnostics can boost early screening efforts, save time, and may improve patient outcomes. The model's performance statistics did not, or only slightly, decrease within six months before medical diagnosis. Our AI-aided blood test interpretation can diagnose most of the analyzed disease groups at least one-to-six or even one-to-nine months before the current traditional clinical diagnosis. A significant decrease in early diagnostic performance was detected only in more acute disease groups, such as liver disease and some cardiovascular pathologies.

Regional and hospital-specific changes and patient behavior (e.g., frequent hospital goers) can impact the time point of possible early diagnosis. The earliest point at which a disease can be diagnosed is also a question of debate, as most of the time, exact thresholds of parameters are to be met for definitive diagnosis. However, we can achieve an earlier diagnosis with more frequent follow-ups for patients in the “predefinitive disease stage”. Furthermore, guideline updates may later include these “predefinitive disease stages” with early treatment options to improve outcomes. Our data suggest that many common diseases may be diagnosed 30–90 days before clinical diagnosis via AI-aided evaluation based on routine phlebotomy results, see Table 4. Our results also indicate that diagnosis earlier than 180 days will be possible in only a few medical cases without the use of other specific blood markers. On the national level, significant savings may come from using AI-aided inexpensive and nonspecific blood test evaluation for early diagnosis; thus, involving AI in laboratory result evaluation may facilitate the much-needed paradigm shift in healthcare toward early diagnosis, treatment, and prevention.

The list of laboratory tests included in our AI analysis covers screening laboratory tests that are commonly used worldwide: basic and complete metabolic panels (ALP, ALT, AST, bilirubin, BUN, creatinine, sodium, potassium, chloride, albumin, total protein, glucose, and calcium); complete blood counts with differential; and screening tests for lipid, iron, hepatic and thyroid metabolism. These tests are among the most frequently ordered tests (28), allowing easy translation of our findings to help efficiently screen a wide range of diseases.

The common laboratory tests used as input are part of routine phlebotomy due to their importance in diagnosing many acute and chronic diseases. For example, TSH tests are widely recommended for first-line thyroid dysfunction screening (29, 30). Moreover, AST, ALP, GGT, and bilirubin levels alone may indicate the correct type and etiology of liver disease, allowing for a targeted investigative approach during clinical examination and further diagnostic testing (31). Although these tests are part of general medical education and common medical knowledge, the clinical interpretations of these tests are far from trivial (31, 32). In addition to diagnosing acute liver pathologies, proper evaluation via liver tests also helps diagnose Wilson's disease; thus, liver tests can distinguish rare pathologies from common medical conditions (31). A comparison of certain blood analysis methods and other factors may also raise issues that are difficult to handle and may lead to misinterpretation or misdiagnosis. For example, sTSH test reference ranges and values are highly sensitive to measurement technology, circadian patterns, analytical platforms, and geographic regions (33). Therefore, following AI-aided analysis, including interpretative comments in laboratory reports, could decrease error rates, thus improving the quality of laboratory information and patient safety (34). Although the major driver for including interpretative comments is new and complex tests in laboratory reports (35), interpretation of common laboratory tests is also welcome. In a survey in the UK, 88% of primary care doctors and nurse practitioners found interpretative comments on the thyroid, gonadotropin, and glucose tolerance test reports helpful (36). AI-supported decision-making may also decrease the rate of missed or late diagnoses originating from HCPs' incorrect interpretation of even basic laboratory tests. Self-reported testing practices for anemia include the overuse of screening laboratory tests, the underuse of bidirectional endoscopy to evaluate new-onset iron deficiency anemia, and the misinterpretation of iron studies, which were also included in our analysis (37). As elaborated in (4, 19–25, 28–32), laboratory testing is a major part of the current SOTA diagnostics for all major common and rare diseases. AI is anticipated to enhance diagnostic accuracy while simultaneously reducing the workload of medical professionals in the interpretation of laboratory results. Fitting AI to the current SOTA should be a focal point of further research and development of tailored protocols for each individual disease.

**Figure 6:**
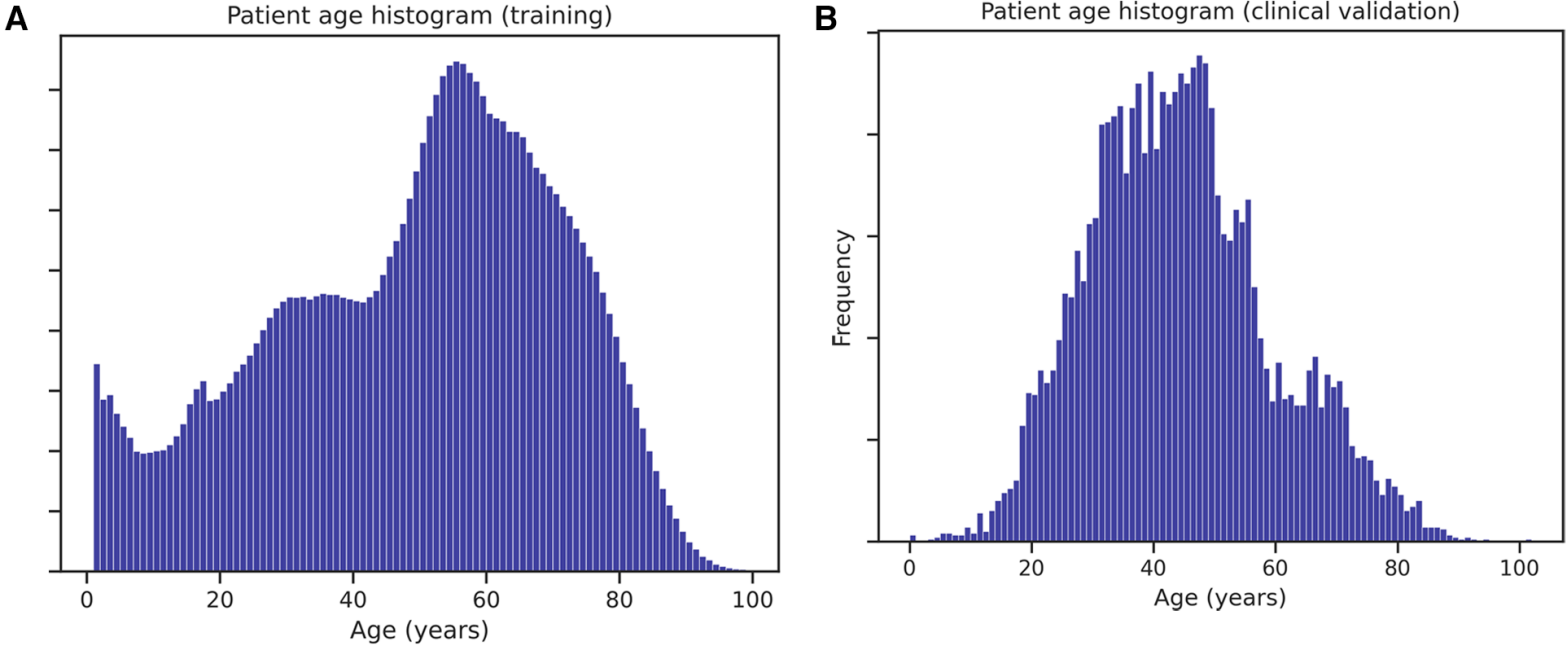
Age histogram of patients in the validation set **(A)** and in the historical data used for training the models **(B)**.

The data validation in a private health clinical environment confirmed the hypotheses and findings on the retrospective model-build data (both with common and rare diseases). Patient age histograms, see Figure 6, and disease prevalences show that the clinical test population and the training population differ significantly. However, the ensemble was robust enough to perform well on the different populations. The differences in disease prevalences can be explained by considering that the training data originated from tertiary and quaternary care hospital data, while the clinical test was carried out in private outpatient healthcare (less complicated cases and generally healthier patients). We did not find a satisfactory explanation for the major discrepancy regarding immune diseases. This has to be further evaluated in the future from both medical and informatic aspects. All participating medical doctors regarded very highly the diagnostic evaluations of the AI. Though the clinical pathologists gave higher scores to the system, the clinical internists agreed more with the individual disease decisions. The differences were significant but not large. This might show differences in approach toward technology among the medical professions, but this has to be further evaluated.

### 5.1 Limitations

Initial results are promising, several limitations must be considered. First, our study's dataset, while comprehensive, was limited to a couple of regional hospital sites. Larger and more diverse datasets from different regions and healthcare settings would provide a better understanding of AI's potential across various demographics and testing platforms.

It is also important to note that we obtained the lowest amount of early diagnostic data (i.e., people's participation in screening programs) for malignancies, a category where early diagnosis is especially critical for effective therapy and where our findings show the possibility of very effective AI-assisted early screening.

Despite the promising results, discrepancies between AI-aided blood test interpretations and current medical diagnoses must be acknowledged. Traditional diagnostic approaches integrate multi-modal data sources (e.g., medical history, physical examination and imaging), whereas our model is limited to laboratory data.

## 6 Conclusion

This study demonstrates the potential of AI-aided decision support systems in disease classification using only routine blood tests, offering valuable contributions to early diagnosis and healthcare efficiency.

Our model is *ab ovo* different from related works (7–13) as our model does not focus on a selected disease but is meant to detect a large number of diseases and conditions. We demonstrated that robust mathematical models and ensemble methods can yield reliable disease classifications across diverse patient populations and laboratory settings. We found that machine learning is strongly capable of recognizing pathological blood test result patterns and integrating AI into laboratory analysis could lead to earlier diagnoses, improved patient outcomes, and a more cost-efficient healthcare system. The novelty of our approach lies in (1) using only routine laboratory tests for AI-based early screening (2) allowing the models to train through the entire patient history timeline (not just on diagnostic “snapshots”) (3) focusing on broad disease categorization to provide medical personnel a generic instead of a specific diagnostic aid, and (4) combining multiple models to create a robust ensemble which proven to work across multiple laboratories and regions. This is a valuable advancement over existing developments, which often focus on more specific diagnostic tests for disease confirmation. However, when comparing existing and recent studies in this field with our method, see Related work, we also see many similarities. For instance, in (12) the authors also used routine blood tests for differentiating acute leukemia subtypes (except for prothrombin time and fibrinogen, which we did not include in most common ones, the blood tests are similar to our initial feature set), and found that the best models were gradient boosting with the similar performance which we reported. We both found MCV, LDH, and MONO# as features of key importance. However, we rigorously focused on not using any other features but blood tests (e.g., age). In (9, 10, 13) the authors used similar machine learning models for classification of bacterial infections, Malaria, Alzheimer's disease and reported similarly high AUC values however these studies were either limited in the size of dataset (10, 13) or their attributes included specific blood tests (9, 10, 13).

Implementation of prescreening with AI can reduce screening costs and improve patient selection for personalized treatments. Moreover, the ability to utilize retrospective data enhances the immediate applicability, offering substantial benefits to both patients and healthcare providers. At the national or payee level, healthcare may become more economical by using AI-aided inexpensive and nonspecific blood test evaluations for early diagnosis. Future work should focus on expanding the dataset to include a broader and more diverse patient population, integrating multi-modal data sources.

## Data Availability

The data analyzed in this study is subject to the following licenses/restrictions: Both source code and source medical data are proprietary technology and can not be shared publicly. However, for clinical and research use, the developed software is accessible free of charge to non-profit medical organizations (using standard HL7 communication of blood test data and outputting standard textual JSON/HL7 with disease/disease group predictions both qualitatively - one and zero - and quantitatively with estimated risk percentages). Therefore, the software may be made fully available based on a request to the corresponding authors or the UDCC Department of Internal Medicine (https://belklinika.unideb.hu/en). Requests to access these datasets should be directed to daroczyb@ilab.sztaki.hu.
