# Supplementary material for: Smart medical report: efficient detection of common and rare diseases on common blood tests
Source: Front Digit Health. 2024 Dec 5;6:1505483. doi: 10.3389/fdgth.2024.1505483 (PMC11656307; doi:10.3389/fdgth.2024.1505483)
Supplement: Supplementary file 1 [file Datasheet1.pdf]

Supplementary material

Appendix A: Machine learning methods

The laboratory tests were assigned to the medical (disease) cases in two different ways. 1) Direct linkage during the diagnostic procedure either automatically (within the HIS) or manually (with rare diseases almost entirely) by an MD during the creation of disease cases (i.e., when a specific blood test result at the beginning of diagnostics resulted in a specific diagnosis). This accounted for 50-60% of all connections. 2) Every laboratory test less than two weeks prior and not more than four weeks after the received diagnosis was directly linked to the medical case. In 40-50% of the cases, the HIS did not provide a tool for automatic association, and manual assignment would have been time-consuming; those tests were not linked. After pairing the laboratory test results with the diagnostic results, we filtered the results according to the most commonly used blood tests based on the department's statistics. We only kept blood tests that are routinely performed for almost all incoming patients. At least one data point must be available for more than 25% of the hospital population in certain spatiotemporal datasets used for training (see Table 2 for more details).

In the development process, we also analyzed the possibility of providing risk assessments for rare diseases. Although each rare disease affects less than one person in 2000 by definition, as there are more than 7000 rare diseases altogether, every 17th person in the population is affected by a single rare disease. This number is already significant; rare diseases often present a unique diagnostic challenge that results in underestimation and underdiagnosis. The involved rare disease groups included 15 rare diseases (conditions), following the Orphanet classification system: rare dyslipidemia (familial hypercholesterolemia, familial chylomicronemia syndrome); rare inborn errors of metabolism (Pompe disease, Wilson's disease, hemochromatosis, Gaucher disease, Gilbert's syndrome, and similar rare conditions of bilirubin metabolism and excretion); rare genetic hepatic diseases (Caroli disease, primary biliary cholangitis); rare hematologic diseases (cryoglobulinemia, hemophilia); rare genetic renal diseases (Bartter syndrome and similar conditions); rare systemic and rheumatologic diseases (adult-onset Still's disease and similar rare autoimmune arthritis); and rare endocrine diseases (Cushing disease, Addison's disease). We included diseases based on the prevalence and expertise within the Clinical Center, where the development process and consultation with rare disease specialists occurred.

Furthermore, as patient data in the hospital information system also included diagnostic codes for malignant diseases, if present, we could train our software on this information and determine whether we could predict the occurrence of malignant diseases in advance. Due to the topic's sensitive nature, this evaluation was made visible only to the healthcare provider, not the patients. The final framework structure is described in Appendix B.

Appendix B: framework structure

The framework package consists of three major parts: a) an ensemble machine learning core, b) an extensive database of laboratory test results, and c) a clinical report generator. The clinical report generator is a technical solution for interpreting the ensemble machine learning core and is not in the scope of the current article. The machine learning core evaluates the risk of the twelve disease groups (thyroid diseases, diabetes and prediabetes,

immunodeficiencies, nutritional and other anemias, liver diseases, gallbladder and pancreatic diseases, kidney diseases, systemic autoimmune syndromes, lipid metabolism disorders, inflammatory bowel diseases - silently - only alerting the physician, not visible for the patient - evaluating the risk of malignant disease group - leukemias, lymphomas, and malignancies of the digestive system) as well as selected rare diseases. The core works as an ensemble of six machine learning models[10] (deep feedforward artificial neural networks, logistic regression, kernel machines, boosting models, elastic net regularization, Bayes network) through the multiple, by a medical team selected single diseases and disease (sub)groups mapped to a zero-one scale where zero means no estimated disease risk and one means the definite presence of disease. Each disease group is split into multiple disease subgroups and single diseases; this multiple-level breakdown is executed based on medical relevance and mathematical performance metrics (the best-performing disease breakdown was also the target of supervised learning) and is summarized in Figure 3. Between the levels and the models, there are weighted consensual (i.e., all models involved have to be supportive) and comparative (i.e., at least one model involved has to be supportive) decisions where the ensemble itself calculates the weights (on which the final decision principally depends) through multiple iterations. Disease grouping follows the ICD-10 conventions: Thyroid diseases (Disorders of thyroid gland) (E.00-E.07), Liver diseases (K.70-K.77), Kidney diseases (N.00-N.19), Noninfective inflammatory bowel diseases (K.50-K.52), Lipoprotein metabolism disorders (E.78), Nutritional anemias (D.50-D.53), Other anemias (D.55-D.64), Diabetes mellitus (E.10-E.14), Systemic autoimmune disorders (M.05, M.30-M.36), Disorders of gallbladder, biliary tract and pancreas (K.80-K.87), Other immune disorders (certain disorders involving the immune mechanism) (D.80-D.89), Cardiovascular disorders (I.20-I.25, I.60-I.89), Leukemias and lymphomas (C.81-C.96), Adenocarcinomas of the digestive system (C.15-C.26).

The disease representation learned by the mathematical models is used to classify the patients into one or multiple disease groups. More details on representation and the machine learning of disease classification can be found in Appendix C. To demonstrate the quality of the learned representation, we show that the patients with different diagnoses align in the space using the UMAP[11] framework (Figure 3a and Figure 3b).

Appendix C: representation learning and classification

Two necessary conditions must be met to efficiently utilize the available laboratory tests:

1. The underlying representation should be readily available and widespread enough to form a representation that can be measured for any potential patient. Therefore, we decided to form the representation based only on widely available blood tests.
2. The representation space and the training data should allow us to develop reproducible models and ensure that all experiments meet the criteria of statistical reasoning.

First, we mapped all cases into a finite-dimensional vector space. We defined the similarity of two cases (indexed by i and j) based on their inner product after normalization:

$$S(i,j) = \sum_{l=1}^{d} \sum_{k=1}^{d} M_{lk} x_{il} x_{jk}$$

where for all i, $x_i \in \Re^d$, x_{il} denotes the l-th coordinate of the vector x_i and $M \in [0,1]^{d\times d}$ is a positive semi-definite matrix determined during preprocessing and based on the data. We used the Sparse Metric Learning algorithm; therefore, before we adopt any classification methods (as described above), we may transform each feature vector based on the optimal metric M: for all i. $x_i \leftarrow M^{\frac{1}{2}} x_i$. All methods utilize this transformed representational space. After applying the transformation to every case related to a single patient and time period, we adopted several methods to classify. Regarding the classification, we considered the following binary classification problem for a single disease. Given the model, we treated every medical case in the training set as an instance. At first, we assigned to each instance the transformed representation, a vector $x_i \in R^d$(in a d real dimensional vector space), and a binary target variable, the label $y \in \{0,1\}$, which is 1 if the patient was diagnosed with the specific disease and zero if not. As we mentioned earlier, multiple conditions can be assigned to a single patient. Therefore, our first ML layer treats diseases as independent conditions; however, the latter steps ensure that the final predictions are robust and consistent by utilizing all results, the individual outputs of the single disease models, coming from the lower layers and models. Since treatment can affect the results, we filtered out every laboratory test dated after the diagnosis of the disease in question if the patient was diagnosed before the date of the laboratory test. This filtering occurs before the vector transformation. We applied six models with various hyperparameter settings based on the final vector space. For the model build, we used scikit-learn 1.2 (https://scikit-learn.org/), Pytorch 2.0.0 (https://pytorch.org/), XGBoost 1.7.0 (https://xgboost.readthedocs.io/), and TensorFlow 2.10.0 (https:// www.tensorflow.org/) software packages, except for kernel calculations and the upper-level ensemble where we also used our own implementation. In the present article, we do not share the exact model weights, the subgroup split where the ensemble performs the best, and the list of feature vector spaces used for training.

Appendix D: evaluation measures

We denoted the correctly classified patients with diagnoses as TP (true positive). Similarly, we denoted the falsely classified patients with diagnoses as FN (false negative), the correctly classified patient without a diagnosis as TN (true negative), and lastly, the falsely classified patient without a diagnosis as FP (false positive). After that we define accuracy (ACC) as the proportion of correctly classified patients in the population

$$Acc = \frac{TP + TN}{TP + TN + FP + FN}$$

while the sensitivity rate (SENS) is the proportion of the correctly classified patients in the population of patients with a specific condition:

$$SENS = \frac{TP}{TP + FN}.$$

False Negative Rate (FNR) is equal to 1-SENS. The ROC curve is defined by the point pairs of true positive rates (TPR or sensitivity) and false positive rates (FPR) at different threshold settings. FPR is equal to 1-specificity, where specificity (SPEC) is the proportion of correctly classified patients within the population of the patients without the specific condition:

$$SPEC = \frac{TN}{TN + FP}.$$

False Positive Rate (FPR) is equal to 1-SPEC. The ROC AUC can be interpreted as the probability of classifying a patient with a specific condition with higher confidence than a patient without the condition. Our final measure was the diagnostic odds ratio (DOR), which can be determined with the parameters mentioned above as

$$DOR = \frac{TP/FN}{FP/TN}.$$

The odds ratio is a valuable measure of test performance, as it is often constant regardless of the diagnostic threshold; however, it is not independent of prevalence[18].

We determined the best models per method by measuring their performance in ROC AUC in a 3-fold cross-validation setup due to high imbalance in certain disease classes (i.e., some target disease classes have an uneven distribution of observations). Although this imbalance can affect the performance of individual models, we need to set the training and test data to be the same for all disease classes in case of evaluating a combination of the methods. Additionally, as expected, the best-performing hyperparameters (e.g., the depth and width of a neural network or the polynomial degree at SVM) were not the same across the disease classes. However, these parameters were similar in the most successful models.
